# Differences in gene expression and variable splicing events of ovaries between large and small litter size in Chinese Xiang pigs

**DOI:** 10.1186/s40813-021-00226-x

**Published:** 2021-09-01

**Authors:** Xueqin Ran, Fengbin Hu, Ning Mao, Yiqi Ruan, Fanli Yi, Xi Niu, Shihui Huang, Sheng Li, Longjiang You, Fuping Zhang, Liangting Tang, Jiafu Wang, Jianfeng Liu

**Affiliations:** 1 College of Animal Science, Institute of Agro-Bioengineering and Key Laboratory of Plant Resource Conservative and Germplam Innovation in Mountainous Region (Ministry of Education), Guizhou University, 550025 Guiyang, China; 2 National Engineering Laboratory for Animal Breeding, College of Animal Science and Technology, China Agricultural University, 100193 Beijing, China

**Keywords:** Transcriptome, Alternative splicing, Ovary, Litter size, Xiang pig

## Abstract

**Background:**

Although lots of quantitative trait loci (QTLs) and genes present roles in litter size of some breeds, the information might not make it clear for the huge diversity of reproductive capability in pig breeds. To elucidate the inherent mechanisms of heterogeneity of reproductive capability in litter size of Xiang pig, we performed transcriptome analysis for the expression profile in ovaries using RNA-seq method.

**Results:**

We identified 1,419 up-regulated and 1,376 down-regulated genes in Xiang pigs with large litter size. Among them, 1,010 differentially expressed genes (DEGs) were differently spliced between two groups with large or small litter sizes. Based on GO and KEGG analysis, numerous members of genes were gathered in ovarian steroidogenesis, steroid biosynthesis, oocyte maturation and reproduction processes.

**Conclusions:**

Combined with gene biological function, twelve genes were found out that might be related with the reproductive capability of Xiang pig, of which, eleven genes were recognized as hub genes. These genes may play a role in promoting litter size by elevating steroid and peptide hormones supply through the ovary and facilitating the processes of ovulation and *in vivo* fertilization.

**Supplementary Information:**

The online version contains supplementary material available at 10.1186/s40813-021-00226-x.

## Summary

Based on analyzing of the transcriptome and alternative splicing events, twelve candidate genes related with fecundity and litter size trait were found out from the ovary of Xiang pig.

## Background

In pig industry, the productive power of sows is one of most concerned economic traits in the world [1]. Reproductive traits are extremely intricate and influenced by multifactors originating from heredity and environment especially in litter size of pigs [2–5]. Lots of genes have functions on the reproduction capability [5, 6]. And number of litter size provides a direct effect on the economic benefits for pig farmer [7]. Ovaries are the main reproductive organs; they perform ovulation and show a direct influence on the efficiency of fecundity. Consequently, the different expression profiles of some important genes in ovary might devote to comprehend the diversity of litter size among breeds [8, 9].

Previous reports focus on the quantitative trait loci (QTL) together with intrinsic genes related with litter size of pigs, and the relationship between genes and the traits [10–12] is explored based on recent technology progress at molecular level [4, 13–15]. Series of genes connected with the fecundity of pig have been found, including *FSH-β*, *ESR*, *OPN*, *MTNR1A*, *PRLR*, *GDF9* and *BMPs* members [16, 17]. Many genes and QTLs have been ascertained to have a linkage with the litter size trait of pig [18–20]. Differently expressed genes related with fecundity and litter size have been detected using transcriptome information from gonads in European pigs [4, 13] and Chinese local breeds [15, 21]. Nevertheless, knowledge of previous works aims at several limited pig breeds and they could not make it clear for the huge diversity of reproductive capability in Eurasian pig breeds.

Xiang pig is one of indigenous breeds in China originated from the southeast mountain environment of Guizhou province. It is featured by short stature, early maturity, excellent environmental adaptability as well as with nice meat quality [22, 23]. Furthermore, the populations among Xiang pig herds present great variation in litter size, ranging from 5 to 21 piglets, while most of sows gave 5–8 piglets per litter from the third to seven parities [24]. It was proposed that the cause might due to the specific regulation in the gene expression related with litter size trait. To screen pivotal genes related with litter size, the ovaries were sampled from two groups of Xiang pigs with large and small litter size. The expression profiles and alternative splicing events of transcripts were analyzed by RNA-seq method. The results will benefit to the interpretation of the molecular regulation manner on the diverse reproduction capability and litter size in pig breeds.

## Materials and methods

### Samples

A total of 40 Congjiang Xiang pigs were prepared from the farm of Dachang pig breeding, Congjiang, Guizhou, China, which born from sows ever giving birth of large litter size as XL group with the total number born (TNB) larger than twelve, or XS group with TNB less than eight. Using the same way from previous reports [15, 25, 26], we randomly chose fourteen pigs that the third estrous time was synchronous from XL group (*n* = 7) and XS group (*n* = 7) to sequence the transcriptome using Illumina next sequencing technology. All sampled pigs were 6 to 6.5 months old, weighing 37.50 ± 3.77 kg with five pairs of nipples. The estrous was first detected by B-ultrasound (KX5000V, XuZhou KaiXin Electronic Instrument Company, China) started from the onset of female standing reflex according to the method reported by Lopes et al. [27]. When the numbers of matured follicles were counted to be 4–8 with diameter larger than 6 mm on one ovary [28–30], both ovaries were picked out by standard surgical operation. The follicles numbers and size on two ovaries were directly measured before putting into liquid nitrogen for total RNA isolation. The sampled pigs were alive and kept feeding under routine method together with other pigs.

### Library construction and sequencing

Based on protocol of Beijing Genomies Institute (BGI), Shenzhen, China, ovarian RNA was isolated with TRIzol method (Invitrogen) with the values of RNA integrity number (RIN) in the scope from 7.9 to 8.8 to prepare cDNA library. The libraries were sequenced using HiSeqTM 2000 platform (Illumina, USA) and generated 100 bp paired-end reads. The same RNA sample was determined in the RNA-seq and qRT-PCR tests. The cDNA libraries together with RNA sequencing were carried out as described previously [26].

### Dataset analysis

The sequencing data from fourteen libraries were taken for analysis of the expression profile and the alternative splicing events of transcripts at estrus stage by RNA-seq method. The raw reads in fastq format were filtered to remove reads under low quality by program Trimmomatic v0.39 using the cutoffs as previous conditions [26], and the clean reads were aligned with the pig reference genomes (Ssc11.1) using HISAT2 software (v2.1.0). The mapped sam files were conversed and sorted into bam format by samtools (v1.1.0). The subread featurecounts software (v2.0.0) was chosen to count the reads amount, which were included in the regions of genes or exons. The expression level of gene was estimated by CPMs values (counts per million mapped reads).

The differently expressed genes with CPM value were calculated by using DESeq2 and edgeR, in which all of CPM values were added 0.001 for logarithm arithmetic. The minimum normalized CPM was 1.0, in which a gene would be eliminated if its CPM value of any sample was not lager than the threshold. The differently expressed genes with CPM values were computed using model featureCounts in subread program (v1.6.3). The threshold for differentially expressed genes (DEGs) was the gene with *P* < 0.001 (false discovery rate (FDR) < 0.005) and log2 ratio > 1 or < -1. rMATS (v4.0.2) was used to detect the differential AS events with an FDR ≤ 5 % and a |∆PSI | |IncLevelDifference| of ≥ 0.1. The value of PSI or ψ (percentage spliced in) value was calculated by rMATs according to the ratio of the long form on total form present to characterize inclusion of exon, differential splice-site choice, intron retention, etc. The DEGs and differentially spliced genes (DSGs) datasets were uploaded to the platform of KOBAS v3.0 (http://kobas.cbi.pku.edu.cn/kobas3) taking reference Sscrofa11.1 as background based on Ontology Consortium (http://geneontology.org/) and Kyoto Encyclopedia of Genes and Genomes (KEGG) for enrichment categories. The ovary DEGs and DSGs from Xiang pig were compared with previous reports [17, 31] via Venn program online. The DEGs and DSGs of Xiang pig related with reproduction were further analyzed. The gene list related with reproduction was first collected from the Gene annotation deposited in NCBI using reproduction as keyword. Then it was converted into the gene names and Ensembl IDs of pig via BioMart online (http://www.ensembl.org/biomart). Based on Venn analysis on the shared genes, the lists of DEGs and DSGs of Xiang pig related with reproduction were selected out.

### RT-qPCR verification

To verify the DEGs and DSGs expression patterns deduced by transcriptome analysis, six samples in each group were used as the same aliquot of total RNA for RNA-seq detection. Seven DEGs (*LDLR*, *SCARB1*, *HSD3B1*, *CYP11A1*, *AKR1C2*, *StAR* and *LRP8*), two highly expressed genes (*SERPINE2* and *RARRES1*) together with five types of AS events were chosen randomly to verify the RNA-seq analysis by quantitative real-time RT-PCR methods. Primers were devised by Primer3 online (http://primer3.ut.ee/). The PCR reaction conditions and proportion were the same as our previous work [32] with each primer concentration of 10 pM/µL, taking *GAPDH* and *β*-*actin* genes as internal controls. Based on dissociation curve analysis for PCR products, the amplification efficiency was controlled within range of 100 ± 10 %. The relative expression level of target gene utilized the method of 2(-ΔΔCt) as reported by Livak et al. [33]. The different level of gene expression between two groups was tested by software SPSS (v21.0) taking the *P* < 0.05 as threshold of significant difference. The results were presented as mean ± standard deviation. The presence of five types of splicing evens were determined by RT-PCR method using pairs of primers outside of the AS region. And the DSGs levels were further qualitatively detected by qRT-PCR method using one primer span both junction ends of AS event. The nucleotide sequences of primers were listed in Table S1.

## Results

### Illumina sequencing and assembly

After quality control, the cDNA libraries of each ovary sample generated 49 ~ 66 million clean reads with 100 base pairs (bp) in length. The fourteen samples showed similar matching results, with mapping ranges of 96.17 ~ 99.96 % onto the genome (Ssc11.1) and 69 ~ 75 % being unique matches (Table 1). The results indicated that all of fourteen libraries present high-quality, together with high percentage of coverage throughout pig reference genome. It enabled us to analyze the transcriptomes profiles from ovary at estrus stage of Xiang pigs with large or small numbers of litter size.

**Table 1:** Overview of RNA-Seq data

Sample	Raw Reads	Clean Reads	Raw bases	Clean Pairs (%)	Total_Alignments (bp)	Coverage (Total_Alignments/ Genome_length)%)	Successfully assigned alignments
XL1	50,011,936	49,981,382	4,501,074,240	24,990,691 (99.94 %)	25,827,924	1.0323273	19,158,796 (74.20 %)
XL2	49,981,708	49,963,086	4,498,353,720	24,981,543 (99.96 %)	25,815,470	1.0318295	18,329,302 (71.00 %)
XL3	50,413,430	50,394,894	4,537,208,700	25,197,447 (99.96 %)	26,161,129	1.0456453	18,061,930 (69.00 %)
XL4	50,375,286	50,340,582	4,533,775,740	25,170,291 (99.93 %)	25,968,916	1.0379626	18,816,287 (72.50 %)
XL5	50,368,172	50,349,522	4,533,135,480	25,174,761 (99.96 %)	26,071,102	1.0420470	18,272,816 (70.10 %)
XL6	52,218,318	50,837,574	7,832,747,700	25,418,787 (97.36 %)	26,311,441	1.0516532	19,834,463 (75.40 %)
XL7	50,004,320	48,770,860	7,500,648,000	24,385,430 (97.53 %)	25,226,371	1.0082835	18,583,326 (73.70 %)
XS1	66,455,060	64,122,488	6,645,506,000	32,061,244 (96.49 %)	32,672,160	1.3058875	22,968,529 (70.30 %)
XS2	66,046,332	63,662,060	6,604,633,200	31,831,030 (96.39 %)	31,595,025	1.2628350	22,116,518 (70.00 %)
XS3	63,770,656	63,770,656	6,377,065,600	30,775,719 (96.52 %)	29,952,835	1.1971976	20,607,551 (68.80 %)
XS4	68,528,104	66,122,768	6,852,810,400	33,061,384 (96.49 %)	33,824,450	1.3519438	25,635,551 (75.79 %)
XS5	66,959,152	64,856,636	6,695,915,200	32,428,318 (96.86 %)	32,875,650	1.3140208	23,289,111 (70.84 %)
XS6	67,665,568	65,344,640	6,766,556,800	32,672,320 (96.57 %)	31,838,098	1.2725505	22,703,748 (71.31 %)
XS7	67,002,792	64,436,586	6,700,279,200	32,218,293 (96.17 %)	31,845,484	1.2728457	22,524,311 (70.73 %)

### Differential gene expression analysis between XS and XL groups

**Fig. 1:**
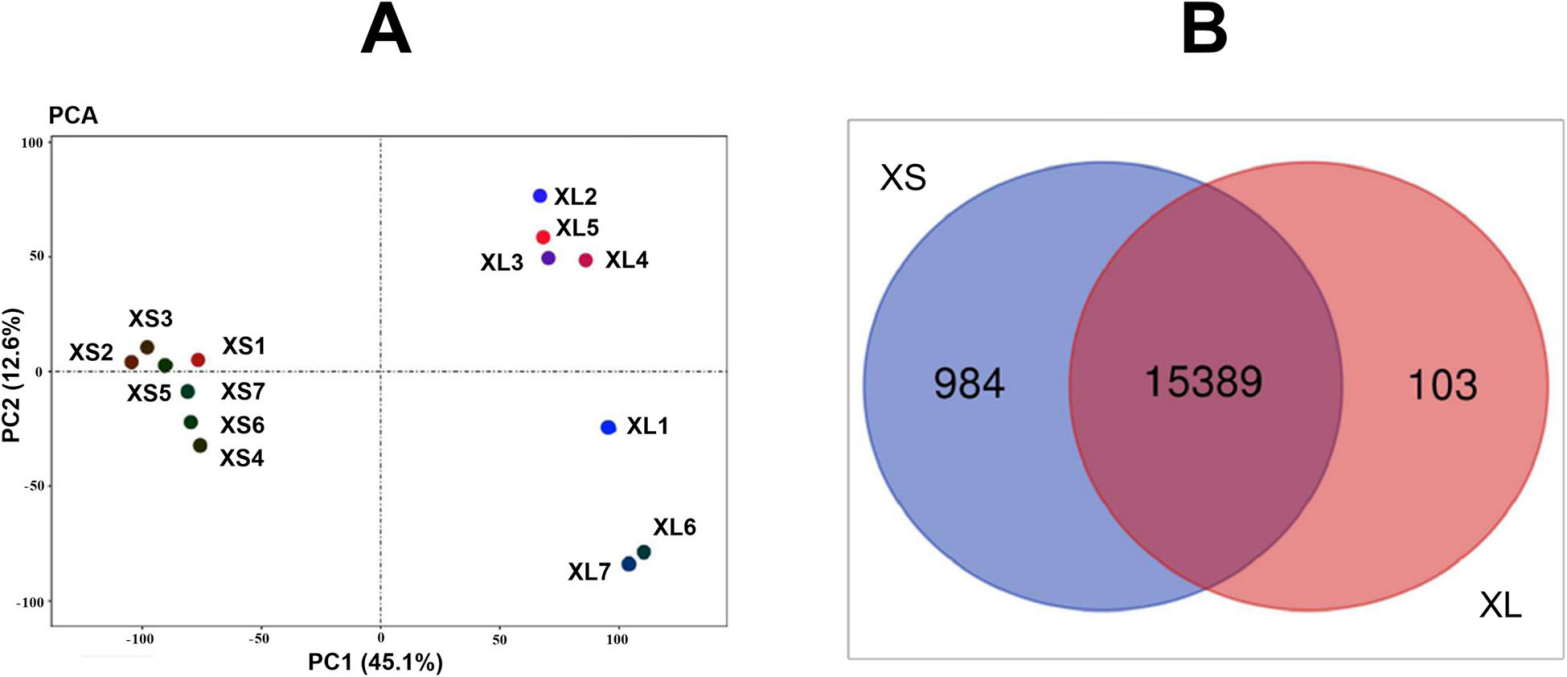
Profiles of gene expression in Xiang pig ovaries between XL and XS groups. **A**: PCA cluster of the gene expression profile of fourteen libraries. **B**: Venn diagram of expression genes in two groups. Blue color represented genes only expressed in XS group, orange colors showed genes only expressed in XL group, and the intersection is the common genes in both groups

Based on mapping data to the reference of pig genome, we obtained 17,089 and 15,928 genes from XS and XL groups, respectively. Normalized CPM data in Table S2 were inputted for principal component analysis (PCA). It appeared that each point in seven samples of the first group could gather and separate from the points in another group according to PC1, which accounted for percentage of 45.1 % of total variation in the dataset (Fig. 1A). It meant that the distance within samples in the same group was much close to each other than that in another group. After removing the noncoding RNA and pseudogene transcripts and those genes with CPM < 1.0 in each sample, the sequencing data of 16,476 genes could be used for following analysis. Of them, 15,389 genes were expressed in both groups; 984 genes were specifically expressed in the libraries of XS group, while 103 genes were determined only from XL group (Fig. 1B). Most of the genes specially expressed in XS or XL group presented in low or very low CPM values. GO analysis indicated that the especially expressed genes in XS libraries enriched mainly in the cellular process, regulation of biological process and metabolic process. However, the genes only expressed in XL libraries had no statistically significant GO terms (Table S3).

**Fig. 2:**
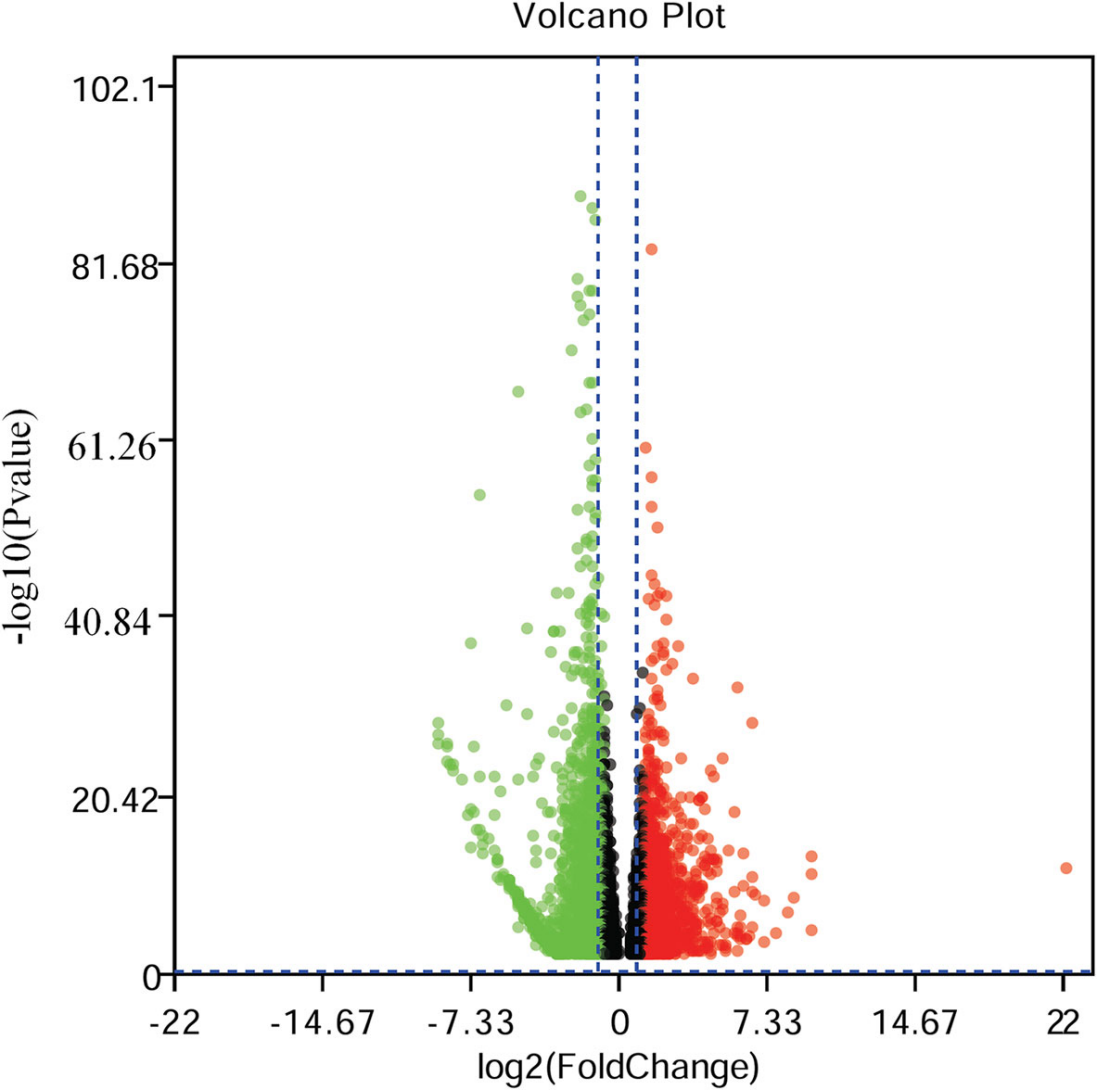
Volcano plots of the differently expressed genes (XL vs. XS). Each point in the figures represented one gene. Red points represented up-regulated genes, green points denoted down-regulated genes. Black points were genes without significant difference

**Fig. 3:**
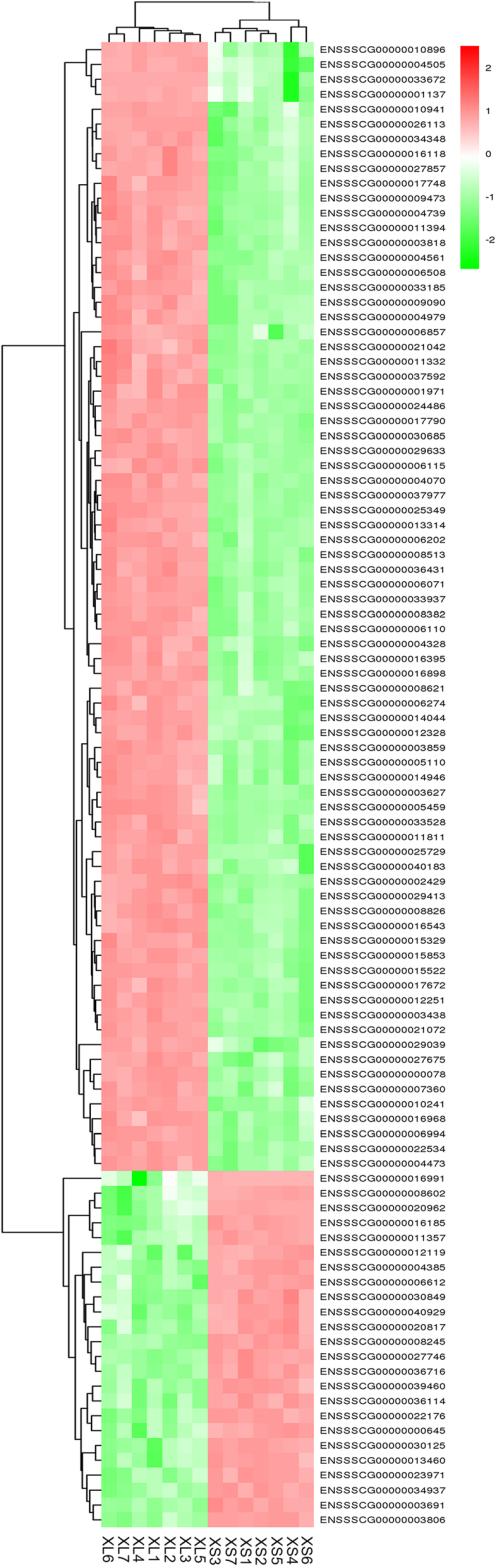
Heatmap showed the most significantly up-regulated and down-regulated genes between XL and XS groups

In total, 2,795 genes were differently expressed between two groups after intersection of results from both softwares of edgeR and DESeq2. Compared with the expression in XS group, 1,376 genes were down-regulated and 1,419 genes were up-regulated in XL group (Table S4). The scope of log2FC values was varied from − 8.75 to 9.31 in DEGs. The numbers of genes, with more than four times of difference between two groups, accounted for 26.11 % of the total DEGs. Approximately 18 and 22 % of the DEGs expressed less than 100 normalized CPM in XS and XL respectively, in which 9.26 % of these genes overlapped between two groups. Moreover, a volcano diagram was plotted based on DEGs data (Fig. 2). The expression levels and numbers of DEGs in XL group were more than that in XS group, as displayed in the heat map of Fig. 3. We compared the top ten genes highly expressed in XS group with that in XL groups (Table 2). The expression level in XL group ranged from 3545.875 ~ 15422.571 CPM (logFC from 0.148 to 6.42), which was decreased to 2594.786 ~ 6588.433 CPM (logFC from − 2.238 to 2.307) in XS group. Of those, the expression levels of five genes (*StAR*, *ATP6*, *COX3*, *COX1*, *SELENOP*) increased and two genes (*MACF1*, *HSPG2*) decreased in XL group.

**Table 2:** The top 10 genes at highly expressed level in XS and XL groups

Gene_ID	Gene symbol	logFC	Pvalue	Padj	Readcount-XL	Readcount-XS	Description
ENSSSCG00000018075	*COX1*	2.307	8.16623E-13	1.26806E-11	15422.571	3116.238	cytochrome c oxidase subunit I
ENSSSCG00000018082	*COX3*	2.607	1.74415E-17	4.91169E-16	6961.009	1142.695	cytochrome c oxidase subunit III
ENSSSCG00000036135	*COL1A1*	0.148	0.521083524	0.609618558	6521.541	5887.012	collagen type I alpha 1 chain
ENSSSCG00000004489	*EEF1A1*	0.531	0.000000096	0.000000671	6455.302	4468.317	eukaryotic translation elongation factor 1 alpha 1
ENSSSCG00000016034	*COL3A1*	0.295	0.169601735	0.244697188	6382.088	5203.422	collagen type III alpha 1 chain
ENSSSCG00000018081	*ATP6*	4.384	8.7737E-25	5.28171E-23	6356.744	304.543	ATP synthase F0 subunit 6
ENSSSCG00000015326	*COL1A2*	0.262	0.300558594	0.392270172	5492.596	6588.433	collagen type I alpha 2 chain
ENSSSCG00000021208	*SELENOP*	1.372	9.40805E-06	4.42317E-05	4865.024	1879.905	selenoprotein P
ENSSSCG00000034942	*StAR*	6.42	3.4925E-30	3.60421E-28	4106.007	47.92	steroidogenic acute regulatory protein
ENSSSCG00000011033	*VIM*	0.362	0.012616959	0.027517822	3545.875	2758.309	vimentin
ENSSSCG00000025675	*EEF2*	0.003	0.970797113	0.977978175	2702.43	2697.277	eukaryotic translation elongation factor 2
ENSSSCG00000003514	*HSPG2*	-1.508	2.12554E-08	1.65259E-07	1779.964	5061.75	heparan sulfate proteoglycan 2
ENSSSCG00000000423		-0.996	0.000106900	0.000398237	1358.776	2709.169	Unknown protein
ENSSSCG00000003654	*MACF1*	-2.238	8.38151E-28	6.894E-26	550.064	2594.786	microtubule actin crosslinking factor 1

More strict thresholds were used to screen out the significant DEGs, which included FDR less than 0.001, the value of normalized CPM larger than 100 and the absolute value of log2FC larger than four. We identified 37 significant differently expressed genes from the total DEGs between two groups, such as *StAR, TIMP1, NR4A1, PTX3, CYP11A1, PTGFR, OVGP1, SERPINE1, CLDN11*, and *MSMO1* (Table 3).

**Table 3:** The 37 DEGs with great fold changes between XS and XL groups

Gene_ID	Gene symbol	Readcount -XS	Readcount -XL	log2FC	Pvalue	Padj	Description
ENSSSCG00000018081	*ATP6*	304.543	6356.744	4.384	8.7737E-25	5.28171E-23	ATP synthase F0 subunit 6
ENSSSCG00000034942	*StAR*	47.920	4106.007	6.420	3.4925E-30	3.60421E-28	steroidogenic acute regulatory protein
ENSSSCG00000025273	*CYP11A1*	95.654	2902.591	4.923	6.4979E-26	4.61379E-24	cytochrome P450 family 11 subfamily A member 1
ENSSSCG00000010400	*MSMB*	29.237	2419.602	6.370	1.84815E-12	2.71985E-11	microseminoprotein beta
ENSSSCG00000012277	*TIMP1*	46.398	2402.269	5.695	2.1928E-34	2.96206E-32	TIMP metallopeptidase inhibitor 1
ENSSSCG00000009759	*SCARB1*	111.493	2083.499	4.224	5.39056E-13	8.60367E-12	scavenger receptor class B member 1
ENSSSCG00000006719	*HSD3B*	124.329	2015.189	4.019	8.923E-14	1.56553E-12	hydroxy-delta-5-steroid dehydrogenase, 3 beta- and steroid delta-isomerase 1
ENSSSCG00000005216	*RLN2*	2.618	1665.513	9.313	1.1897E-12	1.8052E-11	relaxin 2
ENSSSCG00000039548	*PTGFR*	46.064	1086.103	4.559	5.30226E-14	9.52401E-13	prostaglandin F receptor
ENSSSCG00000026724	*PLBD1*	45.017	939.753	4.384	4.86394E-07	2.93018E-06	phospholipase B domain containing
ENSSSCG00000025698	*SERPINE1*	46.572	859.301	4.206	1.66233E-14	3.20042E-13	serpin family E member 1
ENSSSCG00000029066	*IDI1*	40.651	690.156	4.086	8.64835E-15	1.73962E-13	isopentenyl-diphosphate delta isomerase 1
ENSSSCG00000011747	*CLDN11*	33.833	597.989	4.142	9.07434E-17	2.3339E-15	claudin 11
ENSSSCG00000009645	*ADAMDEC1*	23.491	541.132	4.525	1.24429E-14	2.45241E-13	ADAM like decysin 1
ENSSSCG00000008857	*MSMO1*	27.915	479.635	4.102	5.36737E-14	9.63054E-13	methylsterol monooxygenase 1
ENSSSCG00000031321	*NR4A1*	10.930	473.535	5.437	8.68239E-20	3.10901E-18	nuclear receptor subfamily 4 group A member 1
ENSSSCG00000011727	*PTX3*	11.869	423.841	5.158	1.30294E-15	2.87494E-14	pentraxin 3
ENSSSCG00000036956	*SOCS3*	19.526	386.909	4.308	1.15405E-14	2.28814E-13	suppressor of cytokine signaling 3
ENSSSCG00000011683	*PAQR9*	4.394	379.387	6.431	9.82346E-11	1.11946E-09	progestin and adipoQ receptor family member 9
ENSSSCG00000006359	*ADAMTS4*	6.917	327.492	5.565	8.02685E-11	9.22311E-10	ADAM metallopeptidase with thrombospondin type 1 motif 4
ENSSSCG00000003753	*PDZK1IP1*	3.444	324.231	6.556	2.27989E-10	2.45339E-09	PDZK1 interacting protein 1
ENSSSCG00000021576	*CD83*	15.939	270.330	4.085	3.01992E-20	1.15613E-18	CD83 molecule
ENSSSCG00000002013	*DHRS4*	14.232	269.853	4.245	2.07358E-07	1.35533E-06	dehydrogenase/reductase (SDR family) member 4
ENSSSCG00000015268	*FMO1*	11.718	234.182	4.320	1.17801E-12	1.79072E-11	flavin containing dimethylaniline monoxygenase 1
ENSSSCG00000023298	*SRXN1*	10.211	218.387	4.417	4.42571E-24	2.48423E-22	sulfiredoxin 1
ENSSSCG00000009062	*MGARP*	6.837	160.851	4.559	2.91204E-13	4.79996E-12	mitochondria localized glutamic acid rich protein
ENSSSCG00000006791	*OVGP1*	7.036	156.771	4.478	5.01106E-06	2.48682E-05	oviductal glycoprotein 1
ENSSSCG00000008963	*AREG*	0.134	149.218	9.298	9.88649E-15	1.97433E-13	amphiregulin
ENSSSCG00000004521	*MRO*	0.401	145.096	8.434	5.4203E-10	5.46802E-09	maestro
ENSSSCG00000009219	*IBSP*	0.000	111.713	9.293	2.52194E-06	1.32225E-05	integrin binding sialoprotein
ENSSSCG00000028691	*novel gene*	1.780	109.434	5.936	2.32844E-15	5.01127E-14	sulfotransferase 1C1
ENSSSCG00000040843	*MRAP*	5.782	106.633	4.203	1.09225E-08	8.92657E-08	melanocortin 2 receptor accessory protein
ENSSSCG00000034167	*SLC5A3*	476.186	26.881	-4.145	4.07214E-26	2.91632E-24	solute carrier family 5 member 3
ENSSSCG00000026113	*ZBTB20*	197.303	5.575	-5.189	2.60958E-68	2.16791E-65	zinc finger and BTB domain containing 20
ENSSSCG00000033672	*HIST1H1E*	215.637	1.255	-7.490	2.04215E-39	4.03933E-37	histone cluster 1 H1 family member e
ENSSSCG00000034598	*HIST2H2AC*	121.487	0.706	-7.351	2.08391E-27	1.64877E-25	histone cluster 2 H2A family member c
ENSSSCG00000035473	*novel gene*	110.429	0.167	-8.751	1.51499E-27	1.21017E-25	histone H4

### Compare of DEGs between Xiang and Yorkshire pig

Compared with previous report from Yorkshire pig [17], 71 DEGs were found from ovaries of both pig breeds. Of them, 64 genes were up-regulated and 6 genes were down-regulated in the groups with high litter size (Table 4). And six genes, *COX3*, *STAR*, *CYTB*, *CYP11A1*, *MSMB* and *SCARB1*, were ranked in the highest expression level between two pig breeds.

**Table 4:** Compare of DEGs between Xiang and Yorkshire pigs (reported by Zhang et al. [17])

No.	Gene_ID	Gene name	Xiang pig		Yorkshire sow		Gene description
			Up/Down (XL/XS)	Log2FC	Up/Down (YH/YL)	Log2FC	
1	ENSSSCG00000018082	COX3	UP	2.61	Up	2.17	cytochrome c oxidase subunit III
2	ENSSSCG00000034942	STAR	UP	6.42	Up	3.56	steroidogenic acute regulatory protein
3	ENSSSCG00000018094	CYTB	UP	3.09	Up	2.01	cytochrome b
4	ENSSSCG00000025273	CYP11A1	UP	4.92	Up	3.99	cytochrome P450 family 11 subfamily A member 1
5	ENSSSCG00000010400	MSMB	UP	6.37	Up	4.58	microseminoprotein beta
6	ENSSSCG00000009759	SCARB1	UP	4.22	Up	3.59	scavenger receptor class B member 1
7	ENSSSCG00000028512	LDLR	UP	3.78	Up	2.73	low density lipoprotein receptor
8	ENSSSCG00000017164	TIMP-2	UP	1.90	Up	1.65	TIMP metallopeptidase inhibitor 2
9	ENSSSCG00000039548	PTGFR	UP	4.56	Up	3.62	prostaglandin F receptor
10	ENSSSCG00000012583	ACSL4	UP	2.87	Up	2.33	acyl-CoA synthetase long chain family member 4
11	ENSSSCG00000007435	PLTP	UP	3.08	Up	3.21	phospholipid transfer protein
12	ENSSSCG00000013401	DKK3	UP	2.46	Up	2.20	dickkopf WNT signaling pathway inhibitor 3
13	ENSSSCG00000012625	PGRMC1	UP	2.02	Up	2.66	progesterone receptor membrane component 1
14	ENSSSCG00000016267	ITM2C	UP	2.20	Up	1.98	integral membrane protein 2 C
15	ENSSSCG00000011747	CLDN11	UP	4.14	Up	2.77	claudin 11
16	ENSSSCG00000017024	CCNG1	UP	1.59	Up	1.58	cyclin G1
17	ENSSSCG00000018084	ND3	UP	3.87	Up	1.87	NADH dehydrogenase subunit 3
18	ENSSSCG00000010554	SCD	UP	1.95	Up	3.97	stearoyl-CoA desaturase
19	ENSSSCG00000014338	HSPA9	UP	1.02	Up	1.69	heat shock protein family A (Hsp70) member 9
20	ENSSSCG00000036956	SOCS3	UP	4.31	Up	2.90	suppressor of cytokine signaling 3
21	ENSSSCG00000032007	RTN4	UP	1.56	Up	2.36	reticulon 4
22	ENSSSCG00000034207	CEBPB	UP	3.92	Up	2.53	CCAAT enhancer binding protein beta
23	ENSSSCG00000025486	MDH2	UP	1.27	Up	2.16	malate dehydrogenase 2
24	ENSSSCG00000015709	SLC35F5	UP	2.42	Up	2.45	solute carrier family 35 member F5
25	ENSSSCG00000008701	LRPAP1	UP	1.30	Up	1.45	LDL receptor related protein associated protein 1
26	ENSSSCG00000003139	BCAT2	UP	1.65	Up	2.78	branched chain amino acid transaminase 2
27	ENSSSCG00000001770	CTSH	UP	1.47	Up	1.77	cathepsin H
28	ENSSSCG00000022742	PRDX6	UP	1.53	Up	2.52	peroxiredoxin 6
29	ENSSSCG00000027114	SCP2	UP	1.93	Up	5.14	sterol carrier protein 2
30	ENSSSCG00000015268	FMO1	UP	4.32	Up	3.71	flavin containing dimethylaniline monoxygenase 1
31	ENSSSCG00000007739	GUSB	UP	1.29	Up	2.39	glucuronidase beta
32	ENSSSCG00000013599	ANGPTL4	UP	2.89	Up	2.76	angiopoietin like 4
33	ENSSSCG00000009150	HADH	UP	1.24	Up	1.39	hydroxyacyl-CoA dehydrogenase
34	ENSSSCG00000000767	ATP6V1E1	UP	1.04	Up	1.63	ATPase H + transporting V1 subunit E1
35	ENSSSCG00000008237	RETSAT	UP	1.06	Up	1.39	retinol saturase
36	ENSSSCG00000005970	SQLE	UP	2.40	Up	3.42	squalene epoxidase
37	ENSSSCG00000009062	MGARP	UP	4.56	Up	3.66	mitochondria localized glutamic acid rich protein
38	ENSSSCG00000008963	AREG	UP	9.30	Up	5.20	amphiregulin
39	ENSSSCG00000006522	GBA	UP	1.28	Up	1.78	glucosylceramidase beta
40	ENSSSCG00000027130	TNFRSF12A	UP	3.34	Up	1.84	TNF receptor superfamily member 12 A
41	ENSSSCG00000030318	SDHC	UP	1.43	Up	1.83	succinate dehydrogenase complex subunit C
42	ENSSSCG00000000757	ADIPOR2	UP	1.13	Up	1.86	adiponectin receptor 2
43	ENSSSCG00000011723	MME	UP	2.23	Up	2.28	membrane metalloendopeptidase
44	ENSSSCG00000009219	IBSP	UP	9.29	Up	3.41	integrin binding sialoprotein
45	ENSSSCG00000010853	EPHX1	UP	2.21	Up	3.74	epoxide hydrolase 1
46	ENSSSCG00000010537	GOT1	UP	1.48	Up	3.39	glutamic-oxaloacetic transaminase 1
47	ENSSSCG00000032213	DBI	UP	1.30	Up	1.55	diazepam binding inhibitor, acyl-CoA binding protein
48	ENSSSCG00000022998	PKIG	UP	1.74	Up	1.62	cAMP-dependent protein kinase inhibitor gamma
49	ENSSSCG00000006296	ATP1B1	UP	2.15	Up	2.59	ATPase Na+/K + transporting subunit beta 1
50	ENSSSCG00000001435	AGPAT1	UP	1.91	Up	1.42	1-acylglycerol-3-phosphate O-acyltransferase 1
51	ENSSSCG00000006369	F11R	UP	2.76	Up	2.34	F11 receptor
52	ENSSSCG00000016990	ATP6V0E1	UP	1.76	Up	1.81	ATPase H + transporting V0 subunit e1
53	ENSSSCG00000037912	FITM2	UP	1.95	Up	2.20	fat storage inducing transmembrane protein 2
54	ENSSSCG00000006337	HSD17B7	UP	1.69	Up	3.74	hydroxysteroid 17-beta dehydrogenase 7
55	ENSSSCG00000028943	ECH1	UP	1.57	Up	2.16	enoyl-CoA hydratase 1
56	ENSSSCG00000021774	B3GALNT1	UP	1.94	Up	2.28	beta-1,3-N-acetylgalactosaminyltransferase 1 (globoside blood group)
57	ENSSSCG00000034896	HPRT1	UP	1.43	Up	1.50	hypoxanthine phosphoribosyltransferase 1
58	ENSSSCG00000015299	STEAP4	UP	2.61	Up	5.01	STEAP4 metalloreductase
59	ENSSSCG00000009245	SCD5	UP	1.13	Up	1.89	stearoyl-CoA desaturase 5
60	ENSSSCG00000036893	PTHLH	UP	3.24	Up	3.37	parathyroid hormone like hormone
61	ENSSSCG00000006512	FDPS	UP	2.46	Up	3.53	farnesyl diphosphate synthase
62	ENSSSCG00000038221	HSD17B2	UP	5.76	Up	6.95	hydroxysteroid 17-beta dehydrogenase 2
63	ENSSSCG00000007507	PCK1	UP	5.70	Up	6.96	phosphoenolpyruvate carboxykinase 1
64	ENSSSCG00000000182	WNT10B	UP	3.38	Up	2.90	Wnt family member 10B
65	ENSSSCG00000016958	PIK3R1	Down	-1.05	Down	-1.50	phosphoinositide-3-kinase regulatory subunit 1
66	ENSSSCG00000025349	CCDC14	Down	-2.19	Down	-1.49	coiled-coil domain containing 14
67	ENSSSCG00000013772	ASF1B	Down	-1.54	Down	-1.95	anti-silencing function 1B histone chaperone
68	ENSSSCG00000031027	IRS4	Down	-2.02	Down	-3.11	insulin receptor substrate 4
69	ENSSSCG00000005136	IFNE	Down	-3.02	Down	-2.10	interferon epsilon
70	ENSSSCG00000009490	DCT	Down	-1.58	Down	-1.86	dopachrome tautomerase
71	ENSSSCG00000006125	CALB1	Down	-2.37	Up	4.00	calbindin 1

### Detection of DSGs and AS events

Five basic types of AS events were classified, including A5SS (alternative 5′splice site), A3SS (alternative 3′splice site), SE (skipped exon), RI (retained intron) and MXE (mutually exclusive exon). The results showed in Table 5. We identified 63,837/64,075 AS events including splice junctions only (JC) and splice junctions and reads on target (JC + ROT) in 11,414/11,468 genes from XS and XL datasets. Thus, approximately 69 % of 16,476 expressed protein-coding genes were subject to alternative splicing. The numbers of AS events were from 1 ~ 28 events (JC or JC + ROT) in a gene. The highest number of alternative splicing event was found out from gene *ARHGEF7* (ENSSSCG00000009551) with 28 events. SE was the most prevalent AS event, followed by MXE, and RI. Compared with other AS forms, the high frequency of SE indicated that the manner of skipped exon significantly might impact transcription and resulted in various isoforms during gene transcription. A total of 4,009 / 7,441 (JC / (JC + ROT)) significant differential alternative splicing events were identified from 2,763 / 3,936 genes (Table S5). Of these, 542 differently spliced genes (DSG) also exhibited differently expressing (Table S4). Compared with XL group, the number of up-regulated AS events was significantly more than that of down-regulated events in XS group (Table 5).

**Table 5:** The types of AS events in ovaries of Xiang pig with small and large litter size

EventType	NumEvents.JC.only	SigEvents.JC.only	up	down	NumEvents.JC + ROT	SigEvents. JC + ROT	up	down
SE	49,441	1353	643	710	49,634	2325	1723	602
MXE	11,177	3386	1749	1637	11,198	6160	3201	2959
A5SS	622	169	155	14	624	202	191	11
A3SS	837	152	138	14	837	184	173	11
RI	1760	1077	1076	1	1782	1116	1115	1
Total	63,837	6137	3761	2376	64,075	9987	6403	3584

Notes: NumEvents.JC.only: total number of events detected using Junction Counts only. SigEvents.JC.only: number of significant events detected using Junction Counts only. NumEvents .JC + ROT (ReadsOnTarget): total number of events detected using both Junction Counts and reads on target. SigEvents. JC + ROT (ReadsOnTarget): number of significant events detected using both Junction Counts and reads on target

### Compare of DSGs between Xiang and Yorkshire pig

Based on Venn results of the shared genes between Xiang pig and Large White sows [31], 1,597 DSGs from ovaries of Xiang pigs (Table S6) were also detected as much as 2,236 events by using single-molecule long-read sequencing (SMRT) in 39 tissues of Large White sows [31].

### Changes in expression and AS of the reproduction genes between XS and XL

To understand the possible effects of the variations in expression and AS types of the reproduction genes on the litter size trait, crowds of major reproductive genes were picked out and explored the difference in expression pattern and AS of these genes in two groups. The RNA-seq data from 162 genes involved in reproduction processes were listed in Table S7. We found that 22 genes, such as *ESR1*, *ESR2*, *GNRH1*, *FSHR*, *AR*, *GDF5*, *IRS1*, *CCND2*, and so on, were down-regulated, and 33 genes, such as *PTGS2*, *LIF*, *ECM1*, *BMPR1B*, *GPX3*, *C4BPA*, *MMP19*, *MMP25*, *STAT3*, ect, were up-regulated in the XL group. The alternative splicing analysis indicated that 24 / 86 (JC / JC + ROT) significant differential AS events were presented in 42 reproduction related genes. However, of these, only 11 DEGs were also differential splicing, including *AR*, *G6PD*, *ESR1*, *ECM1*, *BMPR1B*, *HEXB*, *STAT3*, *DNMT1*, *C4BPA*, *MMP23B*, and *LIN9* (Table 6).

**Table 6:** The eleven genes related with reproduction harboring differential expression and splicing

No.	Gene name	Gene ID	Event	∆PSI	LogFC	Description
1	AR	ENSSSCG00000012371	MXE-1	-0.253	-1.56	androgen receptor
			MXE-2	0.683		
2	G6PD	ENSSSCG00000025108	MXE	-0.174	1.85	glucose-6-phosphate dehydrogenase
3	ESR1	ENSSSCG00000025777	MXE-1	-0.126	-1.48	estrogen receptor 1
			MXE-2	0.139		
			MXE-3	-0.174		
			MXE-4	-0.112		
			MXE-5	0.364		
4	ECM1	ENSSSCG00000029230	MXE	0.182	3.53	extracellular matrix protein 1
5	BMPR1B	ENSSSCG00000029621	MXE-1	0.152	2.27	bone morphogenetic protein receptor type 1B
			MXE-2	0.441		
6	HEXB	ENSSSCG00000014073	RI	0.209	1.70	hexosaminidase subunit beta
			MXE	-0.132		
7	STAT3	ENSSSCG00000017403	RI	0.096	1.19	signal transducer and activator of transcription 3
8	DNMT1	ENSSSCG00000013659	MXE	0.325	-1.29	DNA methyltransferase 1
9	C4BPA	ENSSSCG00000015662	MXE	-0.275	3.45	complement component 4 binding protein, alpha
10	MMP23B	ENSSSCG00000003351	MXE	0.156	1.88	matrix metallopeptidase 23B
11	LIN9	ENSSSCG00000021310	RI	0.118	-0.81	lin-9 DREAM MuvB core complex component

### Gene ontology and KEGG analysis

To explain the biological effects of DEGs, we carried out GO and KEGG enrichment analysis (Table S3). For the up-regulated genes between XL and XS groups, 59 significantly enriched KEGG pathways were identified (corrected *P*-value < 0.05), including metabolism pathways (carbon metabolism, citrate cycle (TCA cycle), amino acid metabolism, glycerophospholipid metabolism, cholesterol metabolism etc.), oxidative phosphorylation, illness pathways (rheumatoid arthritis, Parkinson disease, colorectal cancer, type I diabetes mellitus, insulin resistance, hypertrophic cardiomyopathy etc.), physiological process related paths (renin-angiotensin system, complement and coagulation cascades, aldosterone synthesis and secretion, bile secretion, cardiac muscle contraction, adrenergic signaling in cardiomyocytes), immune paths (cell adhesion molecules, antigen processing and presentation), five signaling pathways (MAPK signaling pathway, NOD-like receptor signaling pathway, PI3K-Akt signaling pathway, TNF signaling pathway, PPAR signaling pathway). Notably, three pathways, ovarian steroidogenesis, steroid biosynthesis, FoxO signaling pathway, were included in the regulation of steroid hormone and ovary function. The 904 GO terms were enriched in kinds of physiology process. Significantly, 12 GO terms were gathered in reproductive process, female pregnancy, mammary gland epithelium development and proliferation, placenta development, blastocyst development and embryo development.

Meanwhile, the down-regulated genes enriched in two KEGG pathways, which were oocyte meiosis and progesterone-mediated oocyte maturation (corrected *P*-value < 0.05). And the GO terms gathered in meiosis I and meiosis I cell cycle processes. It illustrated that the DEGs between XL and XS groups were important in both function and development of reproductive system and hence were probably to contribute to litter size between two Xiang pig groups.

Very few KEGG paths and GO terms in both the up-regulated and down-regulated DSGs were significant at the level of correct *P* value less than 0.05 (Table S3). It was found that the up-regulated DSGs were enriched in 19 KEGG pathways and 181 GO terms if the threshold was reduced to the *P* value less than 0.05, including autophagy, endocytosis, and lysosome, phosphatidylinositol signaling system, metabolisms such as protein processing in endoplasmic reticulum, lysine degradation, fructose and mannose metabolism, fatty acid metabolism, ubiquitin mediated proteolysis etc. And the down-regulated DSGs could be enriched in 35 KEGG pathways and 219 GO terms including metabolism, growth cell process and reproduction etc. Of those, the GO term of utero embryonic development, and two KEGG pathways (oocyte meiosis, and progesterone-mediated oocyte maturation) were related with reproduction. These results indicated that the DSGs also participated the regulation processes on oocyte maturation and ovary function in pig.

### Candidate genes related with litter size trait in Xiang pig

We performed the Venn analysis on the differently expressed genes, differently spliced genes, the higher expressed genes (CPM ≥ 100), the top expressed genes and the top differently expressed genes between XL and XS groups. Then, we selected these genes that overlapped in three or more datasets for the next GO and KEGG pathway analysis. According to the reported functions in any similar paths that relate to ovarian steroidogenesis, fecundity, pregnant or embryo development, we identified 12 candidate genes (*StAR*, *DHRS4*, *RLN2*, *PTX3*, *HSD3B*, *MSMO1*, S*CARB1*, *COX1*, *COX3*, *SELENOP*, *CYP11A1*, and *NR4A1*) having a linkage with pig reproduction capability and litter size. Of them, eleven candidate genes were identified to be hub genes connected with 12 to 35 genes based on the network relationship of DEGs by using string online platform (Table 7, Fig. S1).

**Table 7:** The detection of eleven hub genes and the protein-protein network

No.	Gene in node1	Combined gene numbers	Genes in the node 2
1	COX1	35	AICDA,ATP6,ATP8,CAT,COX17,COX18,COX2,COX3,COX5A,COX5B,COX6A1,CYCS,CYTB,EXO1,HFM1,HSPA9,HSPD1,MRPS7,MT-ND2,ND1,ND3,ND4,ND4L,ND5,ND6,NDUFS2,NRF1,PNOC,POLD1,RAG2,REEP5,SDHC,SOD2,TCTP,UBC
2	COX3	23	AICDA,ATP6,ATP8,COX1,COX2,COX5A,COX5B,COX6A1,CYCS,CYTB,HIGD1A,MT-ND2,ND1,ND3,ND4,ND4L,ND5,ND6,NDUFS2,PNOC,RPS12,SDHC,SOD2
3	CYP11A1	17	AR,CEL,CYB5A,CYP21A2,DHCR24,DHRS11,FSHR,GNRH1,HSD11B2,HSD17B1,HSD17B6,HSD17B7,HSD3B1,LIPA,SCARB1,SOAT1,STAR
4	DHRS4	20	ACOT4,ALDH1A1,ALDH1A2,ARHGAP11A,CAT,CYB5A,CYTH4,DAO1,ECH1,EPHX2,GSTO2,HSD17B2,HSD17B4,IDH1,KIF4A,PECR,RDH12,RDH16,RETSAT,STX12
5	HSD3B1	20	CYB5A,CYP11A1,CYP21A2,CYP51,DHCR7,DHRS11,FDFT1,FSHR,HSD11B2,HSD17B1,HSD17B2,HSD17B4,HSD17B6,HSD17B7,LSS,MSMO1,SC5D,SQLE,STAR,TM7SF2
6	MSMO1	25	ACAT1,ACAT2,COL6A5,CPOX,CYB5A,CYP51,DHCR24,DHCR7,FDFT1,FDPS,HMGCR,HMGCS1,HSD17B12,HSD17B7,HSD3B1,INSIG1,LSS,MVK,NSDHL,SC5D,SCD,SCD5,SQLE,STARD4,TM7SF2
7	NR4A1	13	AR,ATF3,DUSP1,EGR1,EGR2,FOS,GRASP,MAPK3,NOR-1,PCK1,RPS6KA3,RTN4,VEGFA
8	PTX3	20	ARMC8,ASAH1,C1QA,C1QC,CEP290,CFP,CST3,CSTB,CTSH,CTSZ,F3,GGH,IDH1,NEU1,QPCT,SELP,TIMP2,TNFAIP6,VEGFA,YPEL5
9	RLN	12	ADCY4,ADCY9,ADM,ADORA2B,ADRB1,FSHR,HTR4,KIAA1109,PTHLH,RAMP2,RLF,RXFP4
10	SCARB1	16	ABCA1,ABCG5,APOA1,CD63,CD81,CD82,CYP11A1,EPHA2,HMGCR,LDLR,OLR1,PLTP,PPARA,PPARG,STAR,TSPAN3
11	STAR	17	ADCY9,AR,CYP11A1,CYP21A2,DHRS11,FSHR,GBA,GBI1,GNRH1,HSD17B1,HSD17B6,HSD3B1,SCARB1,STARD4,TSPO2,UGT8,VDAC1

### Tests and verification

**Fig. 4:**
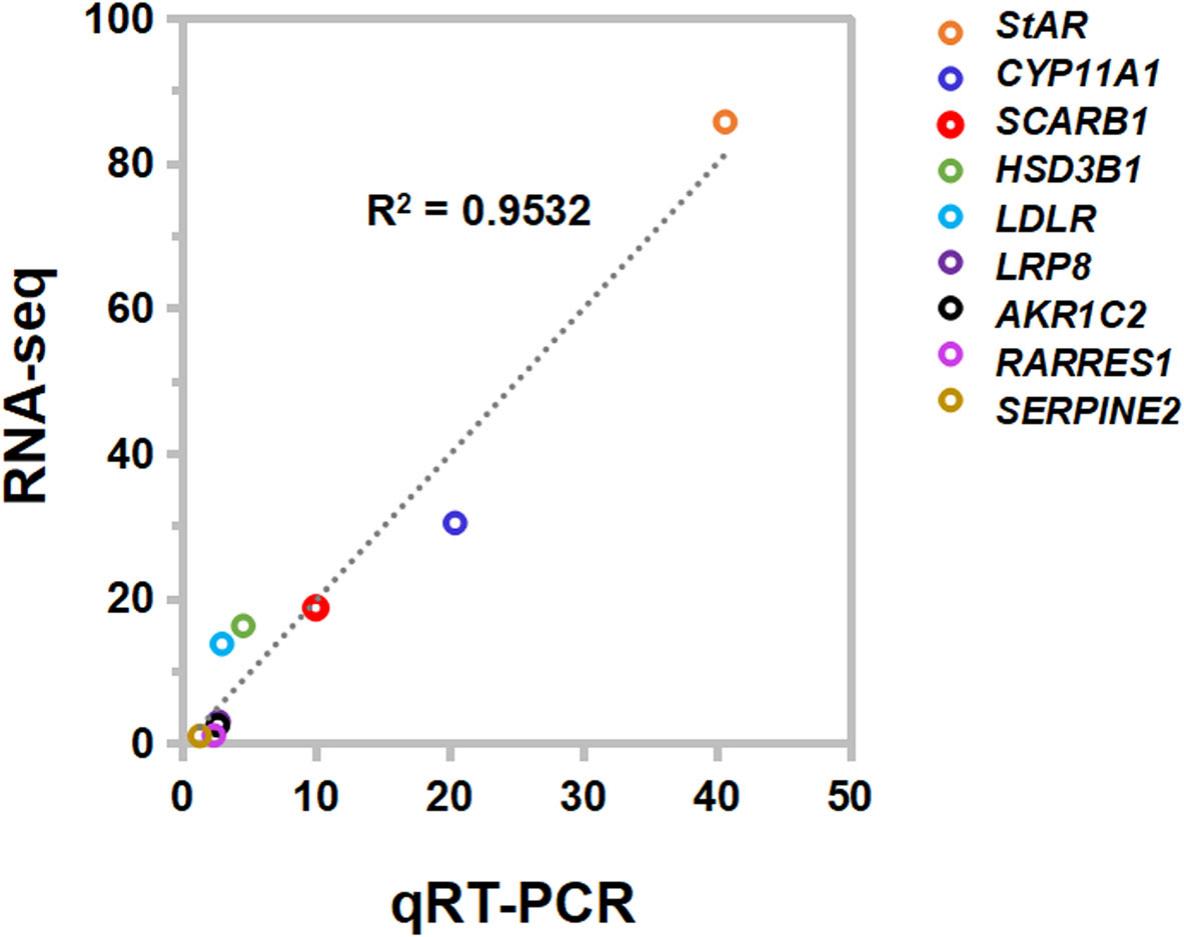
Validation of DEGs by qRT-PCR method. The trend was similar in fold change (XL/XS) from RNA-seq and the ratio of expression levels in groups by qRT-PCR method with the linear correlation coefficient of 0.9532, *P *= 0.037 based on two-tail T-test

The trends of expression of nine genes via qRT-PCR detection were positively related to that from RNA-seq data (Fig. 4). And all of five types of AS events were detected out from the transcripts of ovaries (Fig. S2). It indicated that the analysis based on RNA-seq data was precise and effective.

## Discussion

In this study, we analyzed the estrus ovarian transcriptome and AS of Xiang pig using Illumina next generation sequencing technology. We detected 16,476 genes that expressed in ovaries from libraries. Of these, there were 15,389 genes expressed in common between two groups (Fig. 1B). The expressed amounts of these genes were much diverse, with CPM values changed from 1 to more than twenty thousand (Table S2). Great amounts of genes expressed specifically in XL or XS group detected to be at low or very low level. Further, the top ten genes highly expressed in XL group were compared with that in XS groups (Table 2), and found that the expression levels of some genes increased in XL group, such as *StAR*, *SELENOP*, *COX3*, *COX1*, and so on. The genes with expression level ranked top ten occupied 5.04 ~ 6.96 % of the total expression values in XS and XL groups, respectively. It indicated that these high expressed genes were considerable necessary in the function and the development of ovary. For instance, the transport of cholesterol into mitochondria dependents on the effect of steroidogenic acute regulatory protein (StAR), which accelerates the transform of cholesterol into the inner membrane of mitochondrion to trigger steroidogenesis reaction [34]. In mitochondrion, cholesterol is changed into pregnenolone by the cytochrome P450 side-chain cleavage enzyme. And pregnenolone is further transformed into progesterone or dehydroepiandrosterone, two hormones essential for endometrial receptivity, embryo implantation, and the successful establishment of pregnancy [35]. Previous works indicate that selenium (Se) regulates the growth of granulosa cells together with 17-estradiol synthesis in ovary [36]. Another report showed that both of Se and its selenoprotein (SELENOP), as antioxidants, promote the growth and proliferation of granulosa cells [37].

Previous study for goat ovaries suggests that some special differently expressed genes based on RNA-Seq data may improve litter size [9]. In present work, we identified 2,795 DEGs, including 37 most differently expressed genes between XS and XL groups (Table 3). It indicated that these genes might be very important for the litter size of pig. Results from enrichment analysis indicated that the up- and down-regulated DEGs were clustered in many GO terms and pathways, including metabolism, growth, development, and reproduction. And the effects of the top thirty-seven DEGs between XL and XS groups mainly included ovarian steroidogenesis, metabolic pathways, oxidation-reduction process, negative regulation of endopeptidase activity. Compared with Yorkshire sow [17], 71 DEGs (Table 4) and both pathways (steroid biosynthesis and ovarian steroidogenesis) were shared with Xiang pigs (Table S3). Moreover, 15 genes were up-regulated in the group with high litter size of Xiang and Yorkshire ovaries (Table S3). Some of them are reported to have a pivotal role in ovary. For example, Cyp11a1 protein catalyzes the transformation from cholesterol to pregnenolone in mitochondrion in luteal cells. Both of *StAR* and *Cyp11A1* genes are taken as two marker of corpus luteum in mice [38]. And the *StAR* gene governs the rate-limiting step in steroidogenesis described above [34]. The oxidase HSD3B promotes the oxidation of both delta 5-ene-3-beta-hydroxy steroid and the ketosteroids. The enzyme, 3-beta-HSD, is necessary in the anabolism for all kinds of steroid hormones [39]. As the receptor of HDL, SCARB1 participates in the optional absorption of cholesteryl ether together with the transport outside of HDL-dependent cholesterol, and even accelerates the flow of esterified or free cholesterol on cell surface together with modified lipoproteins [40]. MSMO1 takes part in the first reaction to remove the two C-4 methyl from molecule 4, 4-dimethylzymosterol [41].

Furthermore, the other DEGs listed in Table 3 were reported to have a close connection with ovary function, such as *NR4A1*, *DHRS4*, and *PTX3*. Nuclear receptor subfamily 4 group A member 1 (NR4A1) is an orphan receptor in nucleus, which regulates the transcription of androgen biosynthesis and the expression of paracrine factor insulin-like 3 (INSL3) in thecal cell of ovary. Androgens together with another hormone control the follicle growth in ovary [42]. NR4A1 distributes in many cells of ovary including theca cell, luteal cell, and granulosa cell in human. Furthermore, NR4A1 in Leydig cell is reported to affect the expression of gene *StAR* in mouse [43]. The dehydrogenase/reductase SDR family member 4 (DHRS4) gene, also known as NADPH-dependent retinol dehydrogenase/reductase (NRDR) gene, is a tetrameric protein that is pivotal to the biosynthesis of steroid hormone. DHRS4 functions as NADPH-dependent 3-ketosteroid reductase to produce the 3β-hydroxysteroids from 3-keto-C19/C21-steroids. Types of 3β-hydroxysteroids are reported to transmit signal and participate various physiology functions, such as binding to estrogen receptor β (ER-β) in nucleus and changing the development of prostate [44]. PTX3 is specifically expressed by cumulus cells around oocyte, and mediates the effect of LH or hCG in preovulatory follicle. PTX3 actively participates in the organization of the hyaluronan-rich provisional matrix required for successful fertilization. And PTX3 is taken as a biomarker of oocyte quality and has a role in oocyte maturity and female fertility based on gene deficiency mice [45].

These genes mentioned above, including *Cyp11A1*, *StAR*, *HSD3B*, *SCARB1*, *MSMO1*, *NR4A1*, *PTX3*, *DHRS4* and so on (Tables 2 and 3), were all up-regulated in the ovaries of Xiang pigs with large litter size. It suggested that these genes might play important roles in promoting litter size by increasing the level of steroid and peptide hormones supply through the ovary and facilitating the oocyte ovulation and *in vivo* fertilization.

It is interested that many alternative splicing events from DEGs were detected based on comparison between XS and XL groups. About 69 % of all expressed genes contained AS events in both of XL and XS groups, which is much near to the AS rates in human [46]. Total of 1,597 nonredundant genes with differentially splicing in Xiang pig also detected many isoforms from tissues of Large White pigs [31] (Table S6). In DSGs of Xiang pig, skipped exon was the most prevalent AS events. The rates of AS events in XL group were not as high as that in XS group. AS is the main reason leading to change the different transcripts together with proteome varieties [47]. Numbers of reports indicate that alternative splicing interferes the functions of animal genes and alters the receptor structure especially in the processes of development and growth [48]. Lots of hereditary disease appear strong relationship with high frequency of alternative splicing in genes [49]. It was deduced herein that the high percentage of AS in pigs of XS group might cause the decrease of fecundity. Moreover, we found that 542 DEGs were differently spliced at AS levels between two groups (Table S4). The DEGs presented different and special patterns of splicing and events. Many tops differently expressed genes, such as *StAR*, *MSMO1*, *SCARB1* and *PDZK1IP1* showed high percentages of differently alternated splicing events (Table 2, Table S5). However, there were 3,693 genes only undergoing differently AS events between XL and XS groups. Therefore, the expression patterns and AS events of 162 genes related with reproductive processes were explored profoundly from the RNA-seq datasets. And eleven genes were found to be differently expressed and differently spliced in ovarian samples between XS and XL groups (Table S7). In addition, 31 reproductive genes only underwent differential splicing, such as *CYP19A1* and *FMR1*. Estrogens are essential for animal fertility, which are catalyzed by aromatase enzyme coded by gene *Cyp19a1* [50]. The gene encoding aromatase of mammals contains two promoters, including gonad specific and brain specific promoters. It exists 10 promoters at tissue-specific manner with the first exon to be chosen differently in diverse tissue cells. In kinds of promoters of *CYP19A1* gene, the most vigorous one is promoter II (PII), which drives the transcription of aromatase gene in ovary [50]. The studies from rat find that the expression of aromatase transcription present the diverse and active regulation [51]. Gene *FMR1* (FMRP translational regulator 1; FMRP: fragile X mental retardation protein) is composed of seventeen exons occupying about 38 kb in genome [52]. The gene undergoes extensive AS which changes the retain of four exons, 12, 14, 15 and 17, producing various FMR1 transcript isoforms, and some of FMRP isoforms have been reported in several species [52, 53]. In rat follicles, the *FMR1* gene was transcribed in granulosa cell, theca cell and germ cell. *FMR1* mRNA is much less in pre-ovulatory follicles than that in both preantral and antral follicles. FMRP content raises in the development process of follicles, and could be detected more than four bands by Western blotting method [53]. In present work, the expression of *CYP19A1* mRNA isoform with MXE event was significantly down-regulated and *FMR1* mRNA isoforms with A3SS event were significantly down-regulated in XL group (Table S5). These results indicated that the changes of gene expression between groups with large or small litter size were moderated at many ways and the splicing variants were highly controlled.

Finally, combined with DEGs, DSGs and the higher expressed genes via Venn analysis, we identified 12 candidate genes related with litter size in Xiang pig, including *StAR*, *DHRS4*, *RLN2*, *PTX3*, *HSD3B*, *MSMO1*, S*CARB1*, *COX1*, *COX3*, *SELENOP*, *CYP11A1*, and *NR4A1.* And eleven of them were identified to be hub genes based on the network relationship of DEGs (Table 7, Fig. S1). It indicated that these candidate genes might play important roles in the regulation of reproduction. The effects of *StAR*, *DHRS4*, *RLN2*, *HSD3B*, *MSMO1*, *SCARB1*, *COX1*, *COX3*, *CYP11A1* and *NR4A1* involve in ovarian hormone biosynthesis and regulation, which could regulate the processes of ovary development, oocytes mature and the quality of embryos. The functions of *SELENOP* are related to follicular growth and oocyte maturation [37]. Gene *PTX3* increases the progress of oocyte ovulation and fertilization *in vivo* [45]. These genes expressed at a high level in ovaries of XL group and may accelerate ovarian hormone biosynthesis and the quality of oocytes. They might connect to the higher reproduction performance in Xiang pig. There are eleven candidate genes related with the litter size of Yorkshire breed reported from transcriptome analysis [17]. Interestingly, five of them (*STAR*, *COX3*, *HSD3B*, *SCARB*, *CYP11A1*) were also found to be related with the litter size trait in Xiang pig in this work. But the other candidates from Yorkshire breed are much different from Xiang pigs. It suggested that trait of litter size in pig breeds shared candidate genes and further controlled by diverse genes because of their various genetic background.

## Conclusions

In short, this study showed a transcriptome pattern and AS profiles at estrus stage of ovaries from Xiang pigs. We identified 1,419 genes that showed up-regulated and 1,376 genes that appeared to be down-regulated in the large litter size samples. And it was found that approximately 69 % of expressed genes harbored AS treatment. Of 542 DEGs also exhibited differential alternative splicing. Based on previous finding on those genes, total of 12 candidate genes were found to be corresponding to the reproduction capability and litter size in Xiang pig. These genes play important roles in promoting litter size by increasing steroid and peptide hormones supply by ovary and facilitating the oocyte release and *in vivo* fertilization.

## Supplementary Information

**Additional file 1: Table S1.** Primers for validation of DEGs and DSGs.

**Additional file 2: Table S2.** The expressed genes in the ovaries of Xiang pig with large and small litter size.

**Additional file 3: Table S3.** The KEGG and GO enrichment for DEGs and DSGs of Xiang pig and the compares with Yorkshire pig.

**Additional file 4: Table S4.** DEGs and DSGs between XS and XL groups.

**Additional file 5: Table S5.** The DSG analysis by rMATs.

**Additional file 6: Table S6.** Compare of DSGs between Xiang and Large White pigs.

**Additional file 7: Table S7.** DEGs and DSGs related with reproduction of pigs.

**Additional file 8: Figure S1.** Hub gene networks. **Figure S2.** Confirmation of five types AS events by RT-PCR and qRT-PCR methods.

## Data Availability

The sequencing data is available from SRA database in NCBI with accession number PRJNA737004.
