# Supplementary material for: Differences in gene expression and variable splicing events of ovaries between large and small litter size in Chinese Xiang pigs
Source: Porcine Health Manag. 2021 Sep 1;7:52. doi: 10.1186/s40813-021-00226-x (PMC8411529; doi:10.1186/s40813-021-00226-x)
Supplement: Supplementary file 8 — Additional file 8: Figure S1. Hub gene networks. Figure S2. Confirmation of five types AS events by RT-PCR and qRT-PCR methods. [file 40813_2021_226_MOESM8_ESM.pdf]

Fig. S1 Hub gene networks.

The relationship of candidate gene, *COX1* and *COX3*, with other genes

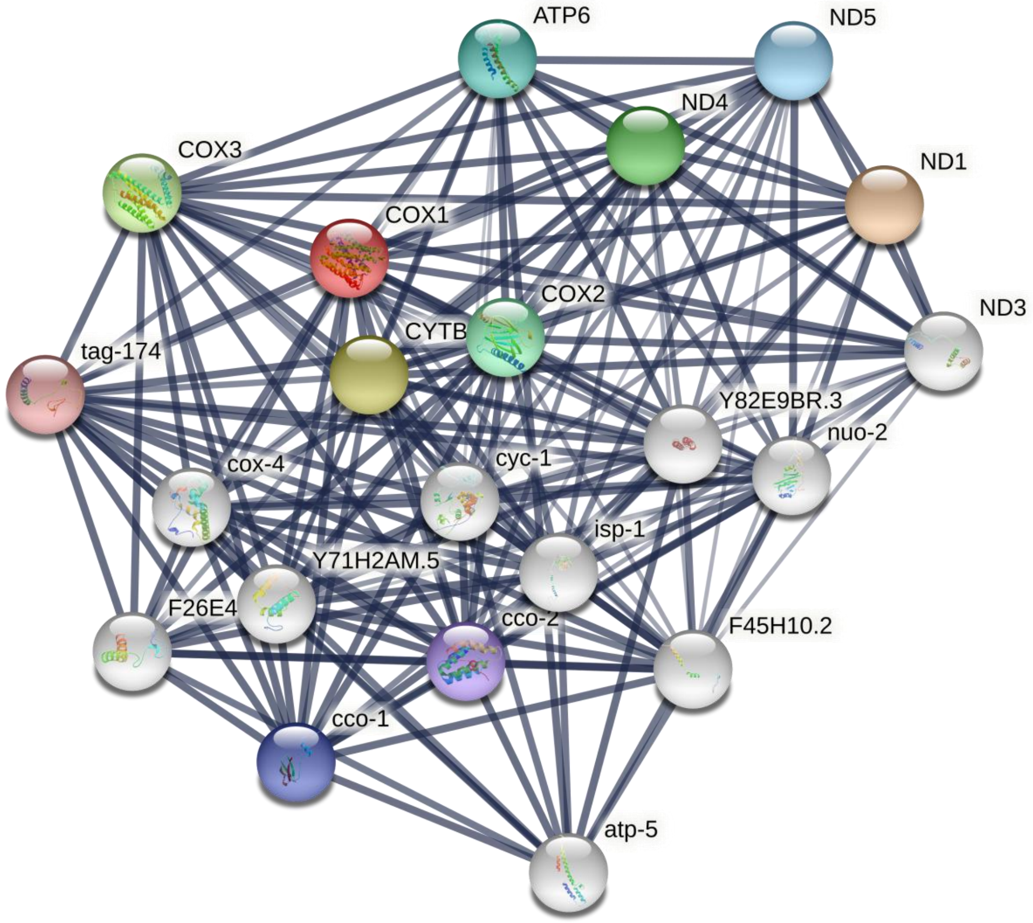

The relationship of candidate gene, *DHRS4*, with other genes

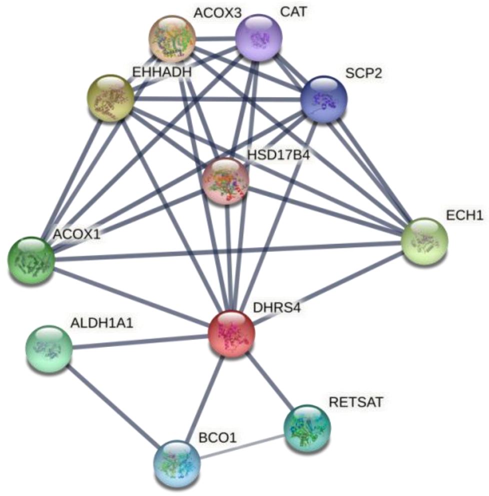

The relationship of candidate gene, *HSD3B*, with other genes

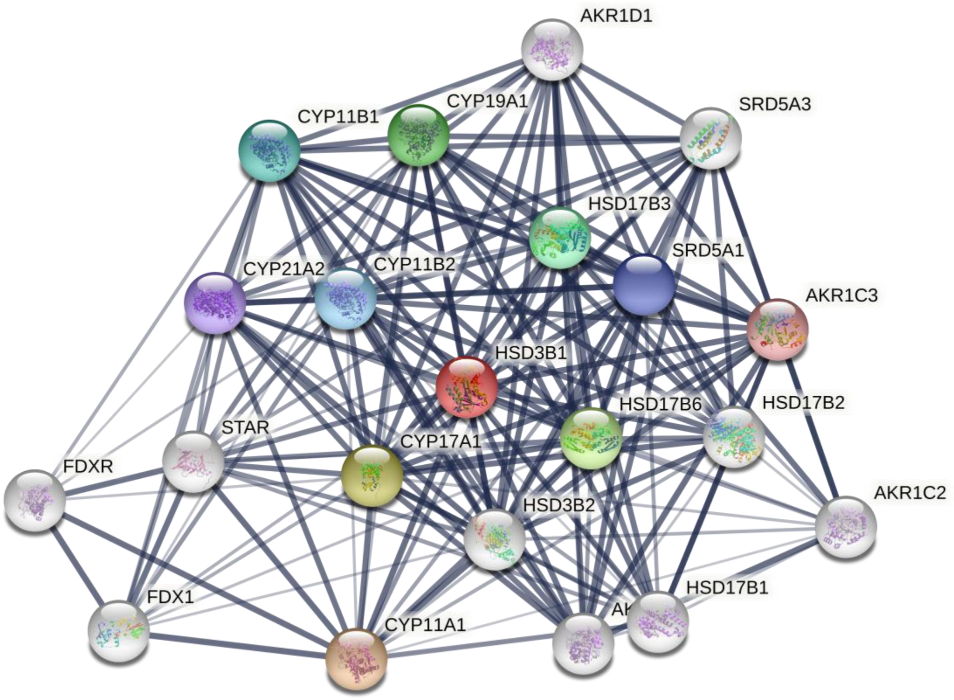

The relationship of candidate gene, *MSMO1*, with other genes

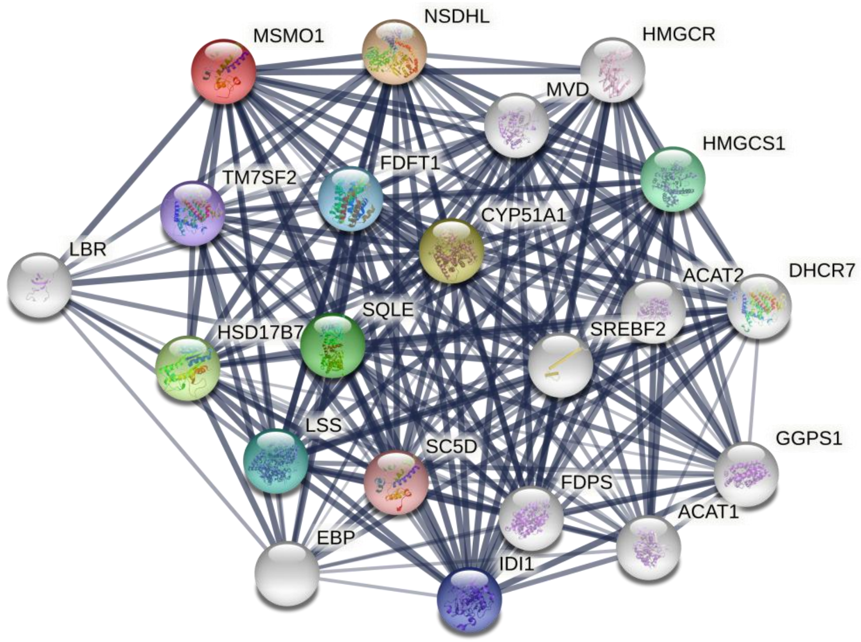

The relationship of candidate gene, *NR4A1*, with other genes

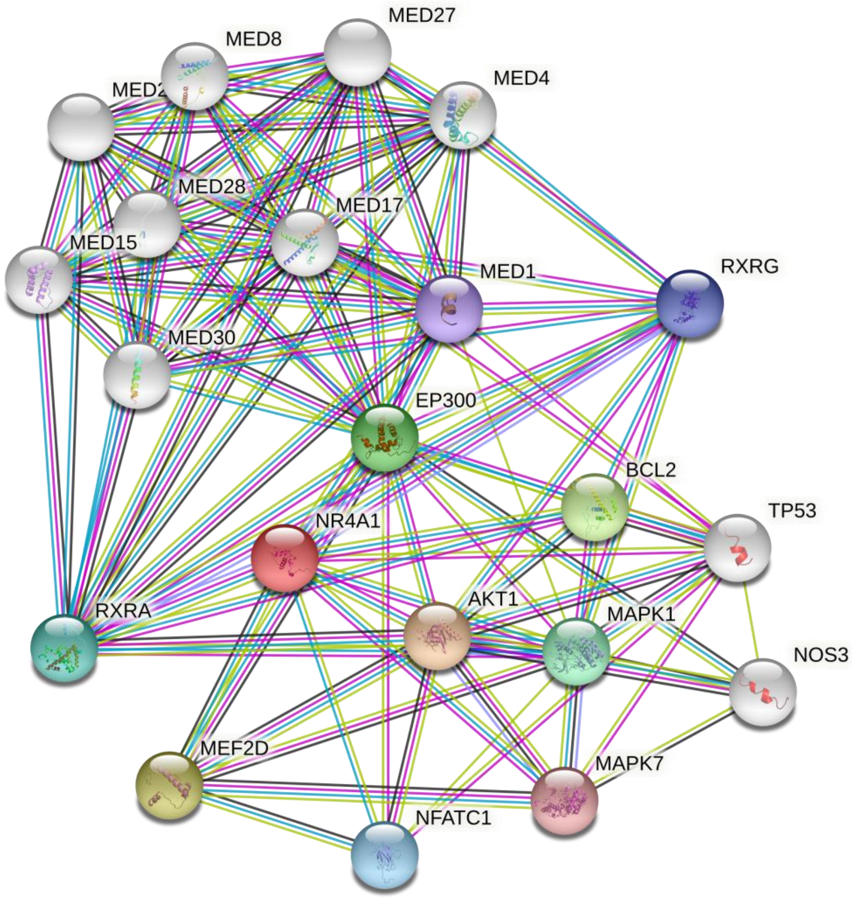

The relationship of candidate gene, *PTX3*, with other genes

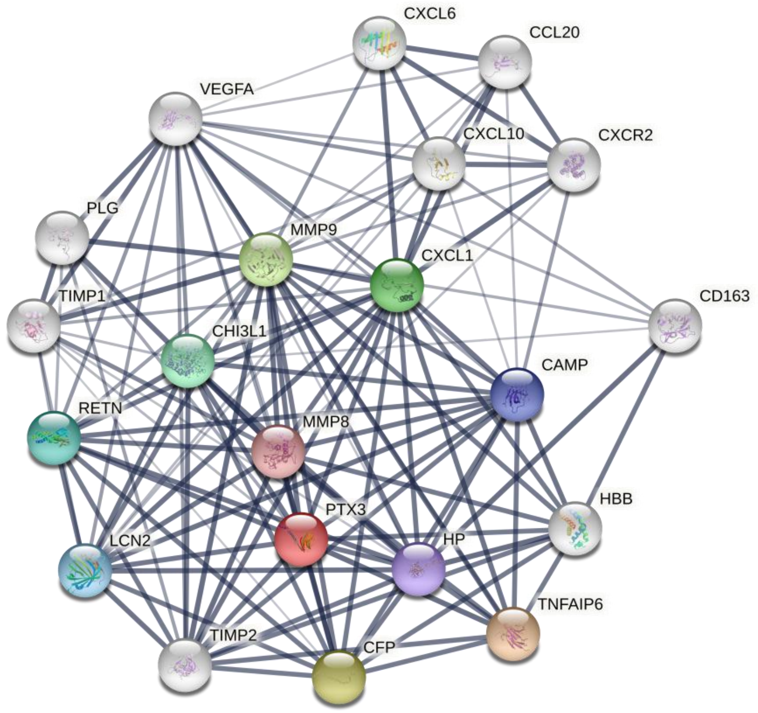

The relationship of candidate gene, *RLN2*, with other genes

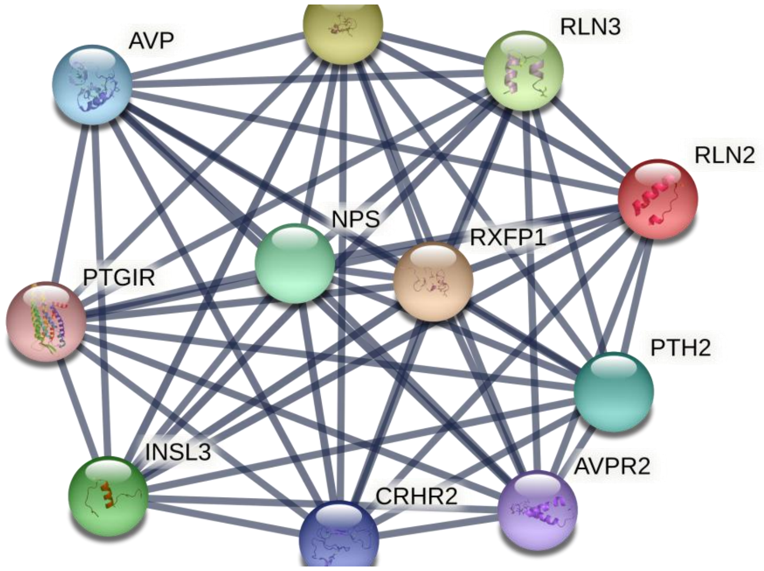

The relationship of candidate gene, *SELENOP*, with other genes

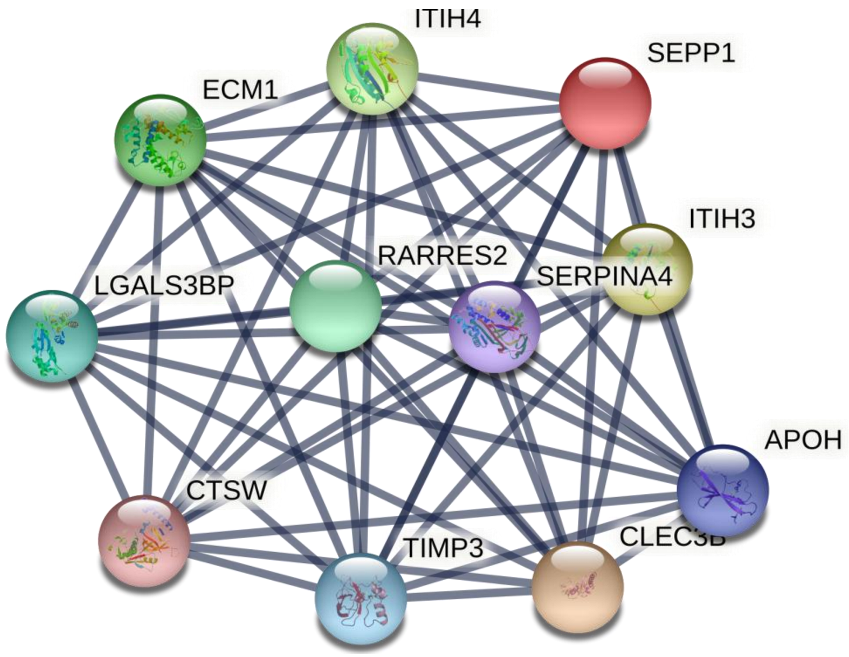

The relationship of candidate gene, *STAR*, with other genes

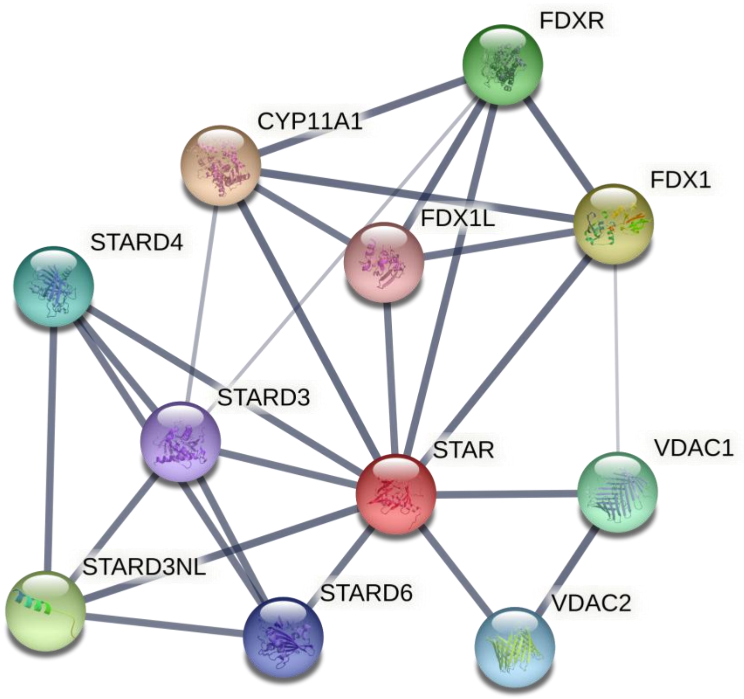

The relationship of candidate gene, *CYP11A1*, with other genes

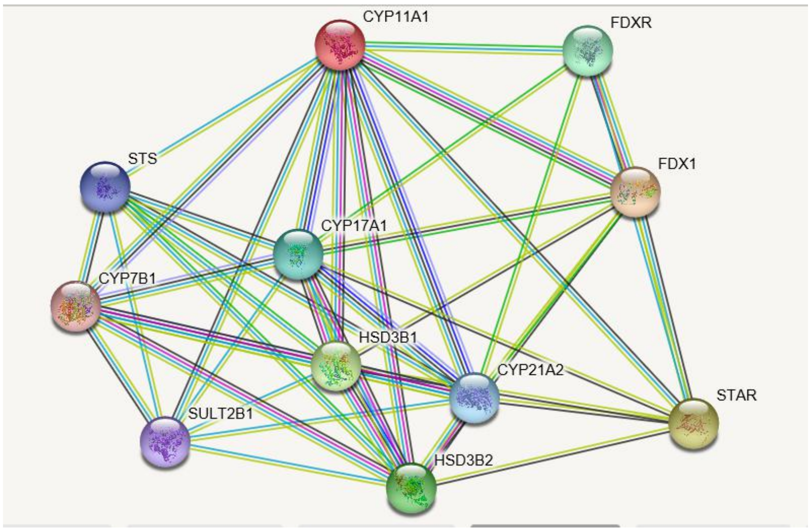

The relationship of candidate gene, *SCARB1*, with other genes

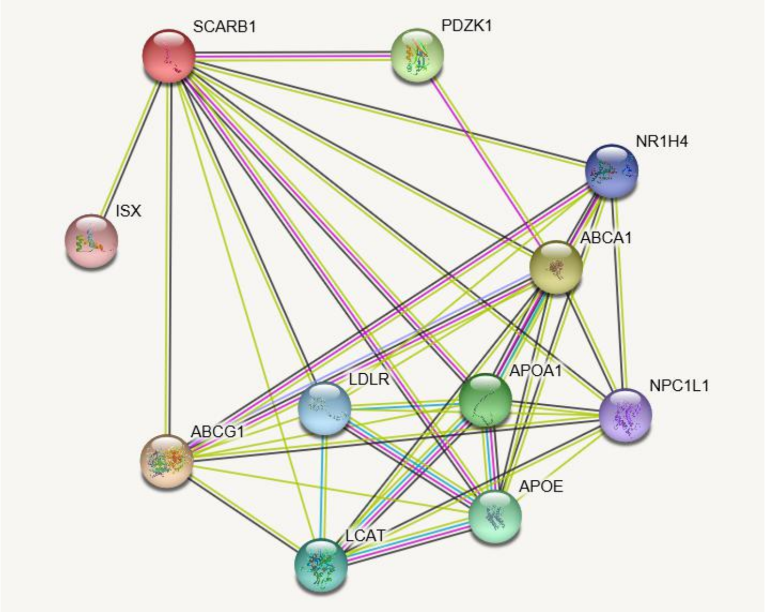

The hub genes for up-regulated DEGs

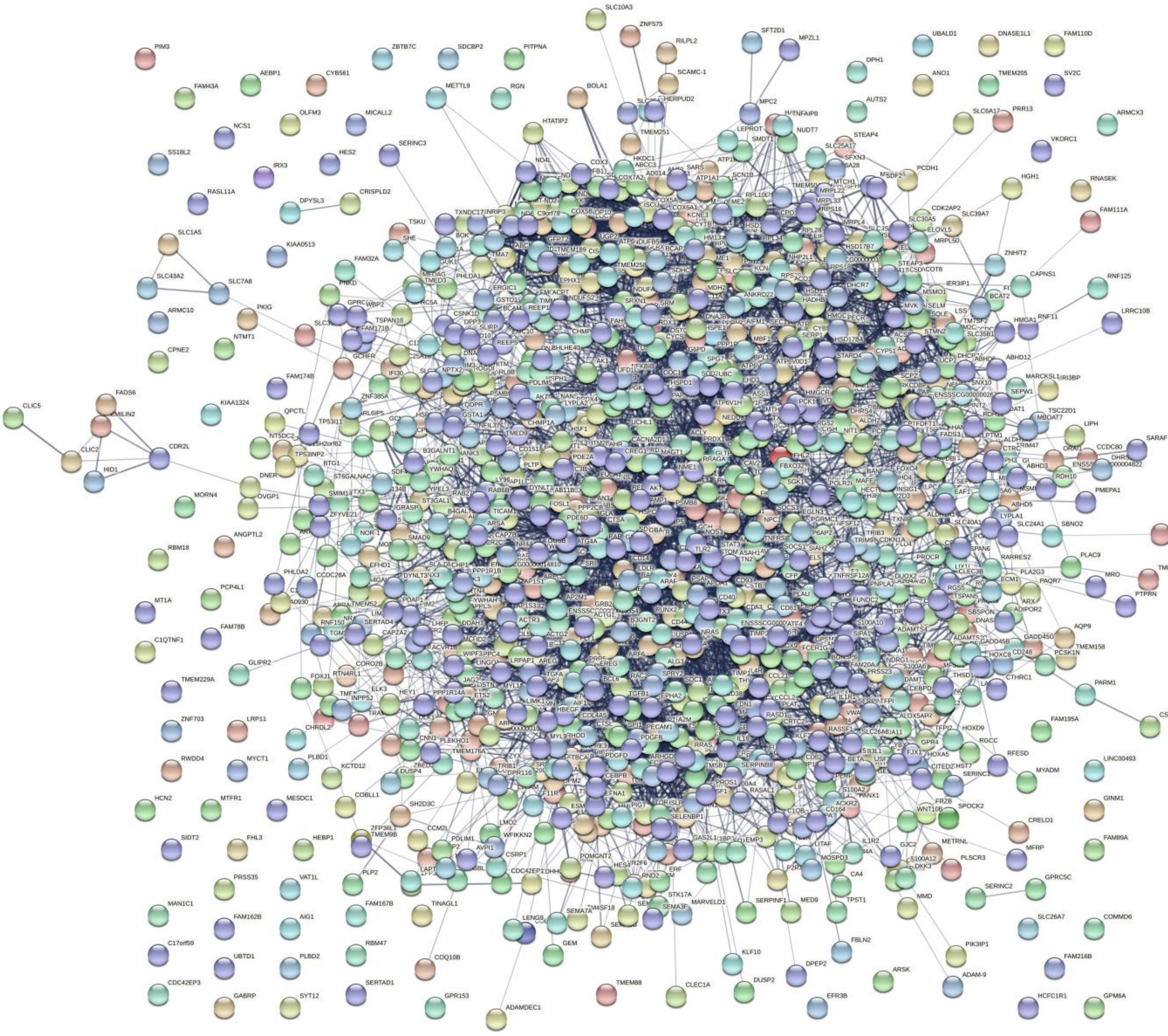

The hub genes for down-regulated DEGs

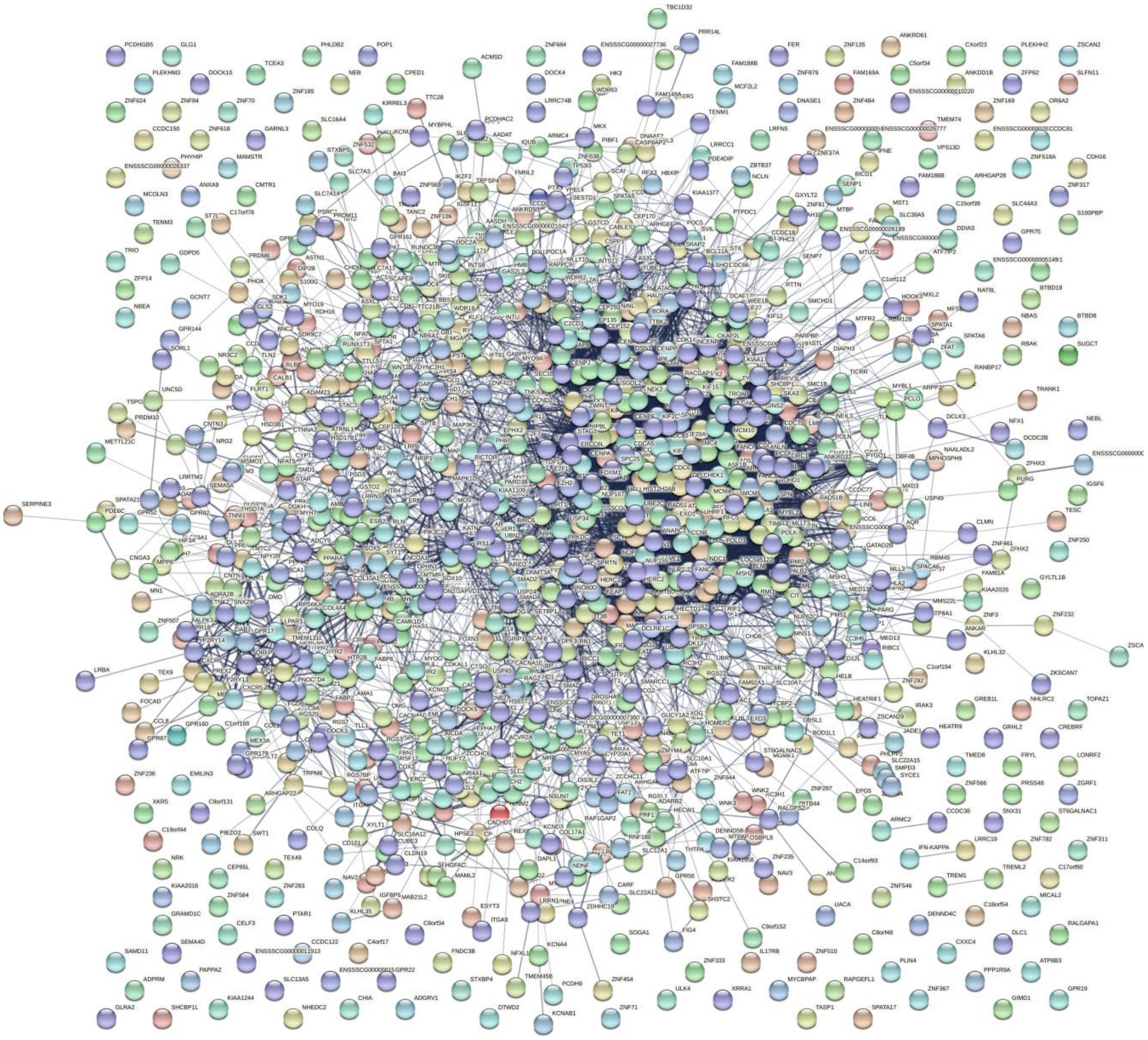

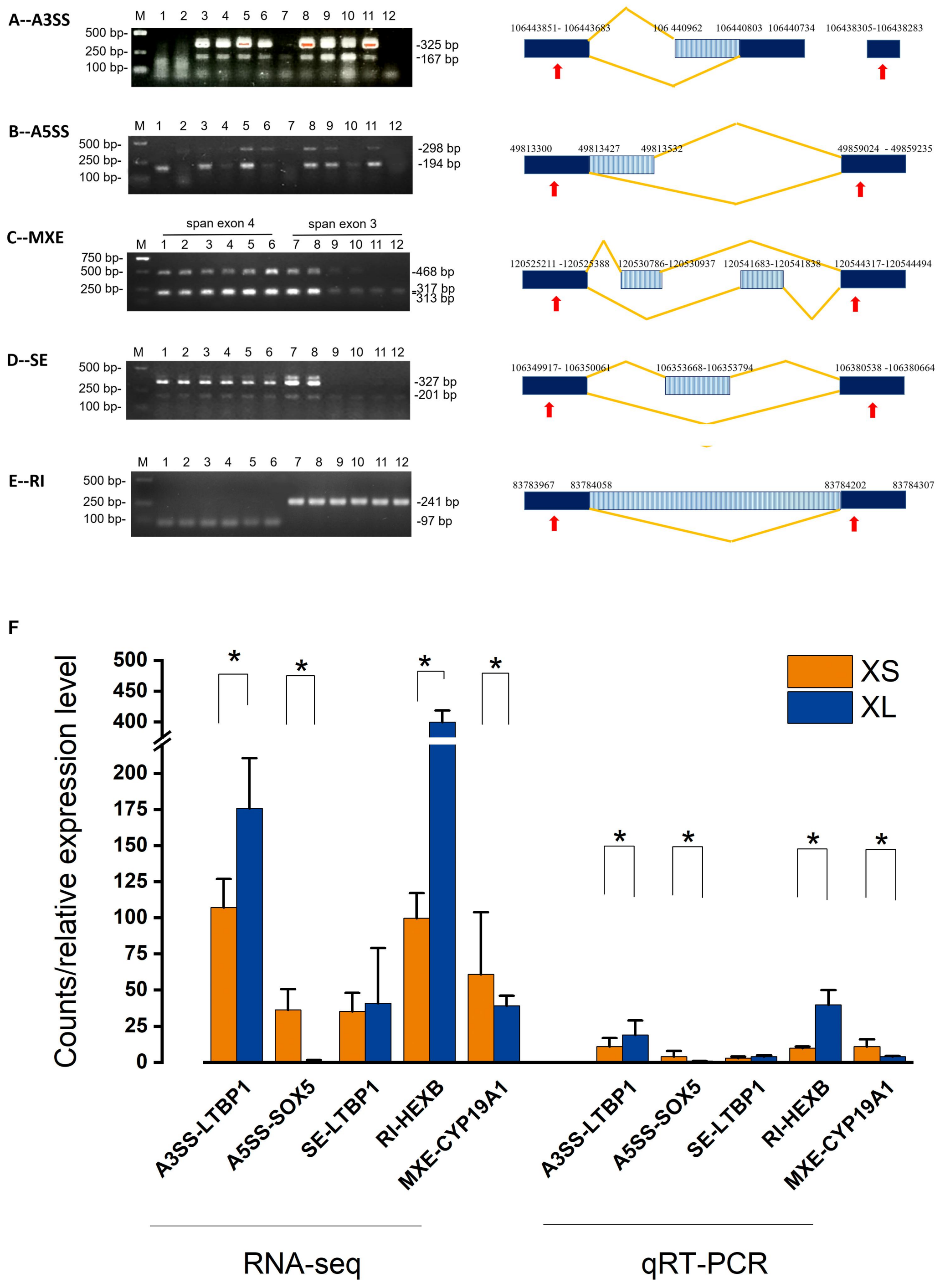

Fig. S2 Confirmation of five types AS events by RT-PCR and qRT-PCR methods.

The five types AS events were verified by RT-PCR method taking the specific primers outside of AS region (panel **A** to **E**), which were detected by 2.5% agarose gel. Diagram of AS types and event locations were arranged on the right. The positions of specific primer were indicated under the exon box in red arrows. **Lane 1-6**: samples from XS group; **Lane 7-12**: samples from XL group. **A**: The A3SS event in exon13 of *LTBP1* were detected with the band of 167 bp in length. **B**: The band with 194 bp in length showed the A5SS event in exon7 of *SOX5* transcript. **C**: Fragments with 313/317 bp in length were the MXE event spanned exon3 (151 bp) or exon4 (155 bp) of *CYP19A1*. **D**: The band in 201 bp denoted the SE event spanned exon27 (126 bp) of *LTBP1*. **E**: The band (241 bp) showed RI event retained in intron 12 (144 bp) of *HEXB*. **F**: The expression level of gene transcripts contained AS events were determined by qRT-PCR with one of primer spanned the junction of AS event listed in Table S1.
